# High-reproducibility, flexible conductive patterns fabricated with silver nanowire by drop or fit-to-flow method

**DOI:** 10.1186/1556-276X-8-147

**Published:** 2013-03-29

**Authors:** Yu Tao, Yuxiao Tao, Liuyang Wang, Biaobing Wang, Zhenguo Yang, Yanlong Tai

**Affiliations:** 1 School of Materials Science and Engineering, Changzhou University, Changzhou, 201326, People’s Republic of China; 2 Department of Materials Science, Fudan University, Shanghai, 200433, China; 3 Department of Biomedical Engineering, University of California Davis, Davis, CA, 95616, USA

**Keywords:** Flexible electronics, Fit-to-flow, Silver nanowire ink, PDMS pattern, Antenna

## Abstract

An unusual strategy was designed to fabricate conductive patterns with high reproducibility for flexible electronics by drop or fit-to-flow method. Silver nanowire (SNW) ink with surface tension of 36.9 mN/m and viscosity of 13.8 mPa s at 20°C was prepared and characterized using a field emission transmission electron microscope, X-ray diffractometer, thermogravimetric analyzer, scanning electron microscope, and four-point probe. Polydimethylsiloxane (PDMS) pattern as template was fabricated by spin coating (500 rpm), baking at 80°C for 3 h, and laser cutting. The prepared SNW ink can flow along the trench of the PDMS pattern spontaneously, especially after plasma treatment with oxygen, and show a low resistivity of 12.9 μΩ cm after sintering at 125°C for 30 min. In addition, an antenna pattern was also prepared to prove the feasibility of the approach.

## Background

Recently, flexible electronics has attracted increasing attention, including batteries, displays [1], conformal antenna arrays [2], radio-frequency identification tags [3], electronic circuits fabricated in clothing [4], and biomedical devices [5], with new characteristics like large area, nonplanar forms, low manufacturing cost, disposable and wearable style, environmentally sustainable production methods, recycling, lightweight, lower energy consumption, and the integration of electronics as a part of other structures [6-10].

Traditionally, etching silicon technology is widely adopted in the microfabrication of conductive patterns in flexible electronics [11-14]. This method involves not only a complicated process but also much pollution. In recent years, many new manufacturing techniques have been improved, such as screen printing [15], gravure [16], inkjet printing [17], dip-pen nanolithography [18], nanoimprint lithography [19], etc.

Though the new technologies have shown great advantages compared with amorphous silicon technologies for flexible electronics, there still exist many problems, for example, some pollution and waste still cannot be avoided during screen printing, printer setups are also very expensive, the defective products produced by these methods are hard to repair, etc. Therefore, more practical technologies need to be studied.

Herein, an unusual strategy was designed to fabricate conductive patterns with high reproducibility for flexible electronics by drop or fit-to-flow method. In this strategy, firstly, silver nanowire (SNW) was synthesized and used to prepare SNW ink. Compared with silver nanoparticle ink, SNW ink provides low sintering temperature and low resistivity, guaranteeing good performance of the conductive pattern, because the continuous conductive track was fabricated by the contact of silver nanowires, not the melt of silver nanoparticles. Though the new emerging organic silver conductive ink can avoid high sintering temperature, but as for conductive track with more narrow line width, there exist many tiny bubbles by this method, resulting in bad performance. Secondly, polymer template (polydimethylsiloxane (PDMS), polymethyl methacrylate, etc.) on polyester (PET) substrate can be easily obtained by spin coating, baking, and laser etching. Thirdly, the prepared SNW ink can flow along the trench of the PDMS pattern spontaneously by drop, especially after plasma treatment with oxygen.

Clearly, compared with the current technologies, the drop or fit-to-flow method shows the following advantages: it decreases the pollution to a lower level and the setups used here are also very cheap. Besides, before the PDMS layer was peeled off, if there exist some defects in the conductive patterns, it can be easily repaired. So, this paper will attempt to describe the strategy. In addition, the feasibility of the approach was also testified by the preparation of an antenna pattern [20-23].

## Methods

### Materials

Silver nitrate (AgNO_3_) was purchased from Shanghai Lingfeng Chemical Reagent Co., Ltd. (Shanghai, China). Poly(*N*-vinylpyrrolidone) (PVP) with molecular weight of about 40,000, ethylene glycol (EG), and CuCl_2_·2H_2_O (99.999+%) were all from Aldrich (St. Louis, MO, USA). PDMS including base and curing agent was obtained from Dow Corning Co. (SYLGARD 184 Silicone Elastomer, Corning, NY, USA). Polyester film (0.1 ± 0.02 mm) was from Shanghai Weifen Industry Co., Ltd. (Shanghai, China). Acetone, ethyl alcohol, and other solvents with analytical grade were got from Sinopharm Chemical Reagent Co., Ltd. (Shanghai, China) and used without further purification. Deionized water was used in all experimental processes.

### Preparation of conductive silver nanowire ink

For a typical synthesis of silver nanowire, a disposable glass vial with 5 mL of EG was suspended in an oil bath (160°C) under magnetic stirring (280 rpm) for 0.5 h. Then, 40 μL of a 4 mM copper(II) chloride solution in EG and 1.5 mL of a 0.147 M PVP solution in EG were injected into the heated EG, followed by 1.5 mL of a 0.094 M AgNO_3_ solution in EG. Then, the color of the solution changed from initially clear and colorless to yellow, to red-orange, to green, to cloudiness, and finally to opaque gray with wispiness, indicating the formation of long nanowires (within 1 to 1.5 h). Silver nanowire powder was isolated from the reaction by centrifugation. The nanowires were washed three times by re-suspension in acetone and centrifugation before use [24].

For the preparation of silver nanowire ink with a solid content of 15 wt.%, the prepared silver nanowire (0.2 g) was re-dispersed by ultrasonic dispersion in a mixed solvent containing 2-butoxy-1-ethanol (0.5 g), isopropanol (0.42 g), and ethanol (0.2 g) to achieve appropriate surface tension and viscosity (36.9 mN/m and 13.8 mPa s at 20°C, respectively).

### Preparation of conductive patterns

For the preparation of PDMS pattern as template, PET was adhered to a sheet glass using double-sided tapes, 3 g PDMS (base/curing agent is 15:1) was dropped on the center of PET film, and then after spin coating (500 rpm), baking at 80°C for 3 h, and laser etching with the power of 5% and speed of 1%, the desired PDMS pattern as template can be fabricated with the conductive track (a thickness of 200 μm and a width of 200 μm) [25,26].

**Figure 1:**
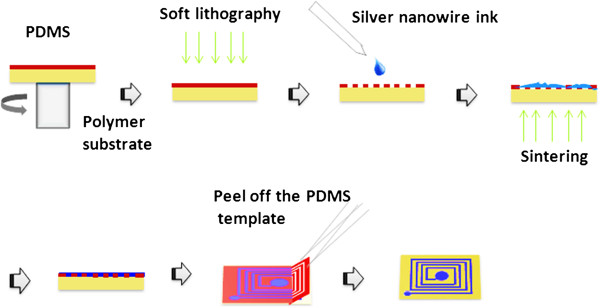
Schematic illustration of the fabrication of polymer-based conductive patterns.

For the preparation of conductive patterns, the synthesized SNW ink was dropped into the trench of the PDMS template track using a syringe, and the ink will flow to all of the tracks spontaneously, till full, then sintered at 125°C for 30 min. Finally, the PDMS template can be peeled off easily using forceps, due to the weak adhesive force between PDMS layer and PET substrate, and the desired antenna pattern was obtained. The details can be seen from Figure 1.

### Instrumentation

The conductive SNW ink and the PET-based conductive patterns were characterized using a Ubbelohde viscometer (CN60M, ZXD Technology Co., LTD, Guandong, China), surface tension instrument (A101, USA KINO Industry CO. Ltd, Valley Stream, NY, USA), transmission electron microscope (TEM; JEM-2100F, JEOL, Tokyo, Japan) operated at an accelerating voltage of 200 kV, X-ray diffractometer (XRD; Max 2550 PC, Rigaku-D, Rigaku, Shibuya-ku, Tokyo, Japan) using Cu Kα radiation, thermogravimetric analyzer (TGA; QS-500, TA Instruments Inc., New Castle, DE, USA) performed in the range of 25°C to 300°C with a heating rate of 10°C min^−1^ in a nitrogen atmosphere, four-point probe (BD-90, Shanghai Power Tool Institute, Shanghai, China), scanning electron microscope (SEM; S-360, Cambridge Instruments, Cambridge, UK) operated at 10 kV, Uscan explorer with 3D profilometer system (D46047, Nanofocus, Oberhausen, Germany), oxygen plasma (PDC001/002, Harrick Plasma, Ithaca, NY, USA), and laser (VLS2.30, 10 W, PPI = 1,000, Versa, Universal Laser Systems, Scottsdale, AZ, USA) with wavelength of 630 to 680 nm.

## Results and discussion

### Properties of conductive silver nanowire ink

**Figure 2:**
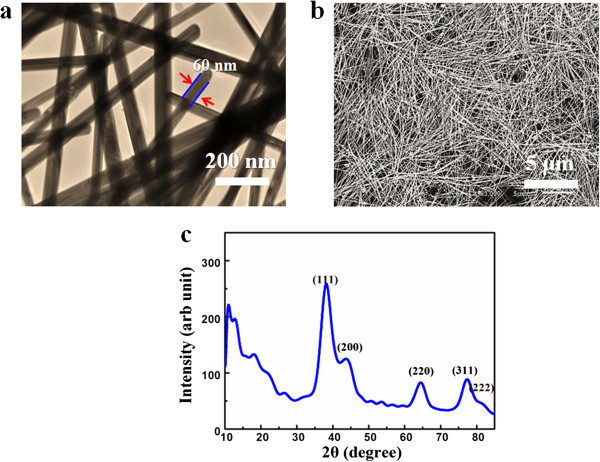
The characterization of the synthesized silver nanowire. (**a**) TEM. (**b**) SEM. (**c**)XRD.

Figure 2a illustrates the TEM images of the synthesized silver nanowire, indicating the uniformity in diameter along each wire with a mean diameter of 60 to 80 nm. This image also suggests that the straightness along the longitudinal axis, the level of purification, and the copiousness in quantity can be routinely achieved through this synthetic approach; the details also can be seen from Figure 2b. Figure 2c shows an XRD pattern of these nanowires, and all diffraction peaks could be indexed to the face cubic phase of silver. The lattice constant calculated from this XRD pattern was 4.098, which was very close to the reported data (*a* = 4.0862, JCPDS file no. 04–0783).

**Figure 3:**
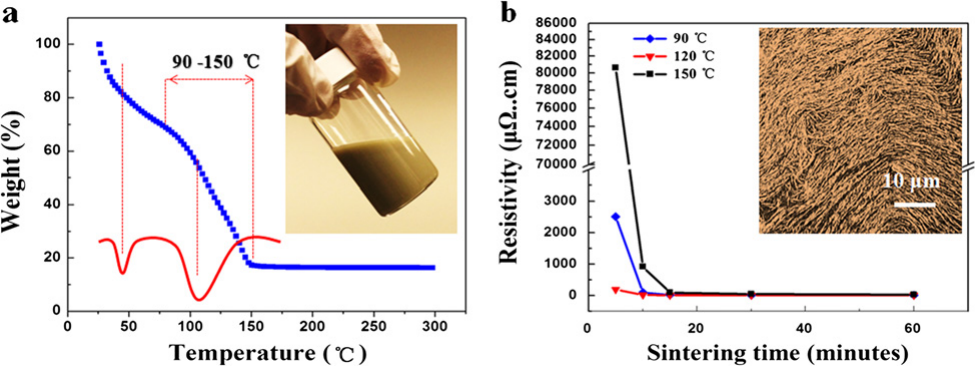
**TGA and DTG curves and conductive properties of silver nanowire ink.** (**a**) TGA and DTG curves (inset, digital image of SNW ink) and (**b**) conductive properties of silver nanowire ink with solid content (15 wt.%) sintered at different temperatures for different times (inset, SEM image of conductive pattern sintered at 125°C for 30 min).

The thermal properties of the prepared silver nanowire ink were investigated by TGA with heating rate of 5°C/min, as depicted in Figure 3a. It can be seen that there exist two mass-decreasing areas, from 30°C to 70°C and from 90°C to 150°C, which are related to the evaporation of low-boiling-point solvents and high-boiling-point solvent and dispersants, respectively; finally, 15.2 wt.% of the mass remains, which indicates that the ink contains 15.2 wt.% silver and agrees well with the calculated value (15 wt.%). The conductive properties of the prepared silver nanowire ink was investigated with different sintering temperatures (90°C, 125°C, 150°C) for different times (from 0 to 60 min), as shown in Figure 3b. During the sintering process, there is no generation of elemental silver like the organic silver ink or melt of nanoparticles like metal nano-ink, mainly up to the solvents and dispersants. Based on the present formula of the ink, when the sintering temperature is 125°C for 30 min, the resistivity can be down to 12.9 μΩ cm.

### Preparation of conductive patterns

**Figure 4:**
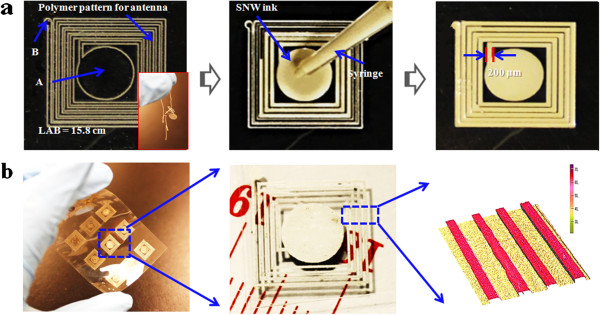
**Fabrication process of an antenna pattern.** (**a**) Fabrication process of an antenna pattern by drop or fit-to-flow method (inset, the peeled off PDMS). (**b**) The prepared antenna pattern after being sintered at 125°C for 30 min and 3D image of the conductive track.

To test the practical applications of the prepared SNW ink and the feasibility of this strategy proposed here, an antenna pattern (11 mm × 12 mm) was designed and fabricated by ink dropping or fit-to-flow method according to Figure 1, which also can be seen from Figure 4a directly.

Figure 4a is the thin-film PDMS pattern template with the thickness of 200 μm, width of 200 μm on PET substrate, and total length of 15.8 cm. The prepared silver nanowire ink was dropped on the center of the template using a syringe (20 μL per drop). Due to the good wetting and film-forming ability of the ink and the hydrophobicity of PDMS template (confine the ink coverage), it will flow along the template track until it fills the whole track, especially after plasma treatment with oxygen. After being sintered at 125°C for 30 min, the continuous conductive track can be fabricated, and the total resistor *R*_AB_ was down to 4.8 Ω measured using a multimeter (Figure 4b), with the width of 200 μm and thickness of 22 μm according to the 3D image, which just was consistent with the solid content of the SNW ink. Therefore, it also can be inferred that the thickness of continuous conductive track can be controlled by the solid content or the layers of conductive track.

**Figure 5:**
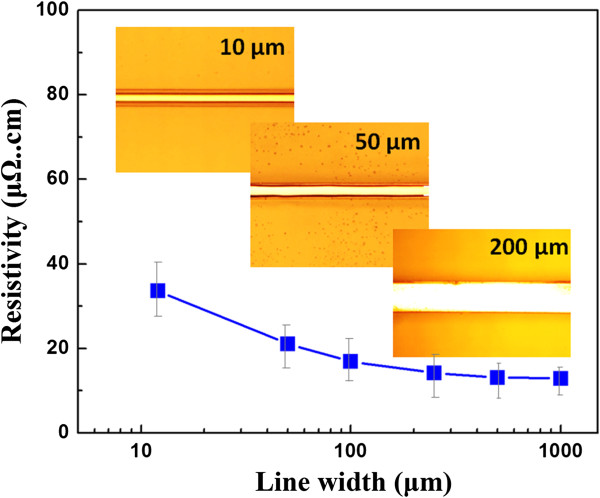
Relationship between resistivity and line width fabricated by drop or fit-to-flow method.

From Figure 5 and inset, a conductive track with different line widths also can be easily obtained by this method. It can be derived that the line width did not have a great effect on the resistivity, and when the line width decreases from 1,000 to 12 μm, the resistivity increased from 12.9 to 33.6 μΩ cm, less than three times, mainly because silver nanowires were as long as tens of microns, as shown in Figure 2b; the alignment of silver wires might be in parallel in a 10-μm trench with less wire crossovers. Therefore, electron transfer might be more difficult. So, it can be inferred that the accuracy of the conductive pattern is mainly up to that of the laser instrument.

## Conclusions

In summary, the strategy of ink drop or fit-to-flow method was applied to prepare an antenna pattern using silver nanowire ink synthesized here successfully. The results show that the SNW ink with the surface tension of 36.9 mN/m and viscosity of 13.8 mPa s at 20°C can flow along the trench of the conductive pattern spontaneously, especially after plasma treatment with oxygen, and showed low resistivity of 12.9 μΩ cm after being sintered at 125°C for 30 min. The relationship between resistivity and line width was also investigated systematically, indicating that this method not only can be used to prepare large-area electronics but also can be fit to the preparation of microelectronics.

## Competing interests

The authors declare that they have no competing interests.

## Authors’ contributions

YT synthesized the silver nanowire and prepared the SNW ink. Y-LT fabricated the conductive pattern and investigated the conductive properties. L-YW, Y-XT, B-BW, and Z-GY gave many advices and took part in writing the whole manuscript. All authors read and approved the final manuscript.
